# High prevalence of syphilis in a female prison unit in Northeastern Brazil

**DOI:** 10.31744/einstein_journal/2020AO4978

**Published:** 2020-05-07

**Authors:** Mara Ilka Holanda de Medeiros Batista, Marcília Ribeiro Paulino, Kaline Silva Castro, Luiz Alcino Monteiro Gueiros, Jair Carneiro Leão, Alessandra Albuquerque Tavares Carvalho

**Affiliations:** 1 Universidade Federal de Pernambuco RecifePE Brazil Universidade Federal de Pernambuco, Recife, PE, Brazil.

**Keywords:** Sexually transmitted diseases, Infections, Syphilis/epidemiology, Women’s health, Prisons, Women, Prisoners, Brazil

## Abstract

**Objective:**

To determine the prevalence of syphilis and the associated risk factors in a female prison unit.

**Methods:**

This was a cross-sectional study including 113 women whom data were collected in two stages: first, blood test to check for syphilis seropositivity; and then collection of information through a form to assess risk situations for sexually transmitted infections.

**Results:**

Overall, syphilis prevalence was found to be 22.1% among the female prison population (n=25) and 28.6% among pregnant women. A statistically significant relationship was found between syphilis infection and previous history of sexually transmitted infections (p=0.04). However, most participants diagnosed with the disease were unaware of a history of sexually transmitted infection in the last 12 months (n=20/80.0%). The use of condom with fixed partners was considered to be a protective factor (odds ratio of 0.76; 95% of confidence interval 0.68-0.85).

**Conclusion:**

The prevalence of syphilis among the female prison population was high, particularly among pregnant women. Preventive and therapeutic measures as well as appropriate prenatal care can minimize the impact of syphilis in prison systems and, consequently, improve such health outcomes nationwide.

## INTRODUCTION

Sexually transmitted infections (STI) have important clinical and psychological consequences for the affected individuals and it remains at high global rates. In 2016, 376 million new infections were estimated such as syphilis, gonorrhea, chlamydia, and trichomoniasis, whose rates varied according to region.^(1)^ Notably, prison systems are reported to have higher rates of STI as compared to the general population. Such condition may be related to risk factors prior to incarceration, including low socioeconomic status, high-risk sexual behavior, and the use of injectable drugs. In addition, other factors taking placing after incarceration may also contribute to STI transmission, namely sexual harassment and socializing with a cluster of people.^(2,3)^

Gathering information on STI inside prison facilities is of great importance, particularly if one considers that imprisonment usually occurs for a short period of time. Hence, the diagnosis and treatment of these diseases could likely cover a considerable portion of the unattended, infected population in the country,^(4)^ besides contributing to reducing the risk of contagion. In this scenario, syphilis has been considered as a prominent disease and is commonly reported in prison systems worldwide.^(2,3,5-9)^

Syphilis is an infectious disease caused by the bacterium *Treponema pallidum*, with complex clinical implications. Its transmission occurs mainly through unprotected sexual intercourses and vertically transmission during pregnancy. Although the main sites of inoculation are the genital organs in general, extragenital areas such as the oral cavity and the anal region may also be affected.^(10)^

In Brazil, 227,663 cases of acquired syphilis were reported from 2010 to 2016, with the Northeast Region ranking the third highest prevalence rates (9.3%). When assessing the proportion of the disease among men and women, there was a decrease in the sex ratio between 2010 and 2015 from 1.8 to 1.5 cases in men for each case in women. Data collected between the years 2005 and 2016 ranked the Northeast Region as the second highest number of reported cases in pregnant women (21.7%). In addition to these figures, between 2014 and 2015, there was a 32.6% increase in acquired syphilis and 20.4% increase in congenital syphilis/pregnant women.^(11)^

The increased incidence of syphilis among women points out to the need for differentiated strategies to prevent infection.^(12)^ When it comes to the female prison population, this category is even more vulnerable and needs special attention and effective actions for disease prevention and treatment. However, the epidemiological characteristics of women’s health in prison systems remain largely unknown.

## OBJECTIVE

To determine the prevalence of syphilis and associated risk factors in a female prison unit.

## METHODS

This was a descriptive, cross-sectional study carried out in 2015 at a Center for Women’s Reeducation in Northeastern Brazil. A total of 343 women corresponded to the prison population in jail, and the study universe was represented by sentenced reeducated women (n=119). Of these, 113 agreed to participate in the study, voluntarily submitting to blood collection for evaluation of syphilis seropositivity.

Tests were performed by the prison unit’s health team in two phases. In the first part of the study, a psychologist provided counseling for rapid tests, and a nurse was in charge of blood collection to check for syphilis seropositivity. Blood samples were drawn by puncturing the digital pulp.

Rapid tests were used to screen for syphilis, namely Rapid Test Dual-Path Plataform (TR DPP^®^; Bio-Manguinhos, Rio de Janeiro, RJ, Brazil). These are single-use screening tests to detect specific antibodies in samples from oral fluid, venous whole blood, digital puncture, or human serum or plasma. The test is based on immunochromatography technology and uses dual-path platform. Reactive results are evidence of exposure to the infectious agent.

The combination of recombinant *T. pallidum* antigens bound to a membrane (solid phase) and protein A conjugate with colloidal gold particles was used to detect disease-specific antibodies. The presence of an antibody is determined based on the appearance of a line in the device, which indicates a reactive reaction.

In the second phase of the study, participants responded to the Quick Test Form proposed by the Ministry of Health on risk situations for STI (http://formsus.datasus.gov.br/site/formulario.php?id_aplicacao=51700). This form enable us to collect sociodemographic data (age, color, marital status, and schooling) and risk factors before and during incarceration, including information related to sexual behavior (type of partner, number of partners, and use of condoms) and drug use (self-use of injectable drugs and partners users of injectable drug). The risk behavior was evaluated based on the association of the study variables addressed in the questionnaire and a positive outcome in the screening tests. Variables possibly related to the seropositivity of the disease were also analyzed.

The data were tabulated using the software (SPSS), version 21.0. Fisher’s exact test was used for comparisons between independent groups, in order to determine possible associations between the variables analyzed and syphilis infection. A p value <0.05 was considered as statistically significant. To estimate the proportion of diseased individuals either exposed or not exposed to risk factors, the odds ratio was verified by calculating the odds ratio, with a 95% confidence interval.

This study had approval of the Research Ethics Committee of *Centro Universitário de João Pessoa* under protocol number 1.34.953 and CAAE: 48845515.6.00005176. Prior to the study, the subjects received a letter of information describing the project proposal, its risks and benefits, and those who agreed to participate in the study signed an Informed Consent Form.

## RESULTS

### Sociodemographic characteristics

The final sample was composed of 113 participants aged 18 and 55 years, with a mean (± standard deviation) age of 29.8±8.2 years. Of the total number of participants, 61 (54.0%) self-reported as mixed race, and 72 (63.7%) were single. Regarding formal education, a total of 78 (69.1%) women had maximum schooling of 7 years (Table 1). Seven pregnant women participated in the study (6.2%).

**Table 1:** Sociodemographic features of the individuals of the study

Variable	Study subjects
Self-reported race	
Mixed race	61 (54.0)
White	37 (32.7)
Black	13 (11.5)
Yellow	2 (1.8)
Marital status	
Single	72 (63.7)
Married/stable union	29 (25.7)
Divorced	10 (8.8)
Widowed	2 (1.8)
Schooling, years	
None	8 (7.1)
1-3	28 (24.8)
4-7	42 (37.2)
8-11	21 (18.6)
12 or more	14 (12.4)

Results are expressed as n (%).

### Syphilis prevalence

Of the 113 study participants, 25 (22.1%) had positive serology for syphilis, which represents almost a quarter of the female prison population in the study. Of the pregnant women, two (28.6%) were diagnosed with the disease. All the results that tested positive for the rapid test were also confirmed by blood test performed at the city’s referral hospital.

### Risk factors for syphilis

The prevalence of syphilis and its association with risk factors are expressed in table 2.

**Table 2:** Prevalence of syphilis and its association with risk factors

Risk factor for syphilis	n	Positive n (%)	Negative n (%)	OR	OR 95%IC	p value
Pregnancy	113			1.44	0.26-7.93	0.48
Yes		2 (28.6)	5 (71.4)			
No		23 (21.7)	83 (78.3)			
Partner	98			0.83	0.26-2.63	0.48
Homosexual		5 (25.0)	15 (75.0)			
Heterosexual		17 (21.8)	61 (78.2)			
Number of sexual partners (last 12 months)	97			0.85	0.33-2.22	0.47
2 or more		12 (24.0)	38 (76.0)			
1		10 (21.3)	37 (78.7)			
STI history last 12 months	113			4.15	1.09-15.72	0.04
Yes		5 (50.0)	5 (50.0)			
No		20 (19.4)	83 (80.6)			
Use of condom/fixed partner	110			0.76	0.68-0.85	0.09
Never/occasionally		24 (23.8)	77 (76.2)			
Always		0	9 (100.0)			
Use of condom/casual partner	90			0.81	0.29-2.23	0.43
Never/occasionally		13 (22.0)	46 (78.0)			
Always		8 (25.8)	23 (74.2)			
Use of injectable drugs	113			3.62	0.21-60.11	0.39
Yes		1 (50.0)	1 (50.0)			
No		24 (21.6)	87 (78.4)			
Use of non-injectable drugs	113			0.88	0.36-2.14	0.48
Yes		12 (21.1)	45 (78.9)			
No		13 (23.2)	43 (76.8)			
History of partners users of injectable drugs	113					
Yes		5 (33.3)	10 (66.7)	1.95	0.59-6.35	0.21
No		20 (20.4)	78 (79.6)			
Frequent consumption of alcoholic beverages	113			1.99	0.72-5.49	0.13
Yes		19 (26.0)	54 (74.0)			
No		6 (15.0)	34 (85.0)			

* Statistical test: Fisher’s exact test; statistically significant at p<0.05. 95%CI: 95% of confidence interval 95%; OR: odds ratio; STI: sexually transmitted infections.

A statistically significant relationship was observed between syphilis and history of STI in the last 12 months (p=0.04). The data revealed as risk factors: history of STI in the last 12 months (OR=4.15; 95%CI: 1.09-15.72); use of injectable drugs (OR=3.62; 95%CI: 0.21-60.11); frequent consumption of alcoholic beverages (OR=1.99; 95%CI: 0.72-5.49); relationship with partners’ users of injectable drugs (OR=1.95; 95%CI: 0.59-6.35); and pregnancy (OR=1.44; 95%CI: 0.26-7.93). The use of condom was considered a protection factor for relationships with fixed partners (OR=0.76; 95%CI: 0.68-0.85) and with occasional partners (OR=0.81; 95%CI: 0.29-2.23). Heterosexual relationship (OR=0.836; 95%CI: 0.266-2.63) and no more than one partner (OR=0.856; 95%CI: 0.33-2.22) were also observed as protective factors. For all the mentioned factors, a 95% confidence interval was considered.

When describing the risk factors of the 113 participants, we observed that 50 (44.2%) of them had sexual intercourses with 2 or more partners in the previous 12 months; and 20 (17.6%) women reported having had homosexual relationships. As for the use of condom, the data show that 101 (91.8%) women had never used or had used occasionally a condom with fixed partners; and 54 (65.5%) of them reported having never/occasionally used condom with casual partners. These findings demonstrate that a large proportion of these women were at risk of being infected with STI.

It is also worth noting that 24 (23.8%) of the participants who had had sporadic or continuous unprotected sexual intercourse with their fixed partners were diagnosed with syphilis. Among the women who reported always making use of condom, none developed the disease.

As for issues related to drug use, 57 (50.4%) reported having already used non-injectable illicit drugs. The use of injectable drugs, on the other hand, was reported by two participants (1.7%). When asked if their partners were injectable drug users, 15 (13.3%) answered yes. The consumption of alcohol was a common practice (n=73; 64.6%) among participants.

## DISCUSSION

The findings observed in our study through rapid tests detected a high prevalence of syphilis, which was almost a quarter of the study population. This shows the importance of awareness campaigns in prisons on disease prevention, detection and treatment. Of note, the lack of knowledge and untreated infections may contribute to the increase in the incidence of syphilis in the general (non-prison) population. Marques et al.,^(3)^ also emphasize that the return of the formerly imprisoned individual to the community likely contributes to the increased risk of disease exposure in the general population.

Syphilis has been detected in several prison units worldwide, with prevalence rates ranging from 0.7% to 11%.^(2,3,5-9)^ For instance, in the State Prison of Guanajuato in Mexico, a disease prevalence of 0.7% was observed,^(5)^ whereas in a Portuguese prison rates were found to be as high as 6.0%.^(3)^ A study in the Venezuelan prison system found a syphilis prevalence of 6.1%.^(9)^ In Ghana, prevalence rates were slightly higher (11%).^(5)^ In Brazil, a study conducted in Pernambuco identified a prevalence of infection of 3.92%.^(2)^ These rates were generally attributed to factors related to sexual life and use of injectable drugs.

Only few studies in the literature have investigated the prevalence of syphilis among imprisoned females. These data are extremely important, since in Brazil, between the years 2014 and 2015, there was a significant increase in cases of syphilis affecting women.^(11)^

The study by Javanbakht et al.,^(13)^ described the reality of a female prison unit in Los Angeles, United States, reporting a syphilis prevalence of 1.4%. In Brazil, these figures are higher, as pointed out in a study carried out in the female prison of São Paulo city, with a prevalence of 5.7%.^(14)^ By investigating the reality of pregnant women imprisoned in Brazil, Domingues et al.,^(15)^ identified a prevalence of syphilis of 8.7% in this population. Our study gathered more current data suggesting that such an issue is actually worsening, with prevalence rates as high as 22.1% among females and 28.6% among pregnant ones.

A study with pregnant women tested positive for syphilis confirmed the presence of complications in some mothers, fetuses, in addition to neonatal syphilis. Thus, the authors emphasize that the disease onset during pregnancy deserves a lot of attention and that its diagnosis is essential for an effective treatment to prevent vertical transmission.^(16)^ In a retrospective study, Lafetá et al.,^(17)^ observed that syphilis is commonly diagnosed at a late stage in pregnant women, either after childbirth or after the curettage process, therefore concluding that prenatal and neonatal care need to be restructured.

In our study, one of the most alerting risk factors was the sporadic use of condoms by the majority of participants. This careless habit perhaps represents the main factor contributing to the increased incidence of the disease in Brazil. Educational actions need to be implemented at an increasing pace, with the aim of reducing the risk of STI contagion and incidence.

Cross-sectional studies have the limitation of not determining a cause-and-effect relationship. Likewise, we also emphasize the need for multicenter studies to better represent the reality of the country.

A number of variables may potentially explain the high frequency of syphilis, including low socioeconomic status in northeastern Brazil; scarcity of prevention-oriented educational activities in prisons; and the low investment in this population’s healthcare. According to Domingues et al.,^(15)^ there is a higher prevalence of syphilis among imprisoned women than in the general (free) female population, in addition to lower quality of prenatal care and high social vulnerability.

Another relevant question is related to the type of test used to detect the disease. In a previous study, Sato et al.,^(18)^ evaluated a rapid test based on the immunochromatography technique for the detection of antibodies against *T. pallidum*. The authors observed sensitivity and specificity values of 93.7% and 95.2%, respectively. Thus, the authors suggest that the test may be applicable as a screening tool and should not be used as an exclusive criterion in the diagnosis of infection. With this, the lack of complementary tests to confirm the data can be considered a limitation, which, however, does not minimize the nature of the findings reported herein.

Today, the implementation of disease-fighting initiatives is often ignored by government officials due to lack of information. Thus, the present study may contribute with data essential for the planning of activities, thereby reducing the public health burden related to syphilis in prisons in Northeastern Brazil and throughout the country.

## CONCLUSION

There is a high prevalence of acquired syphilis among the female prison population, especially pregnant women, which reinforces the need for urgent measures to reduce disease breakthrough and transmission in prison systems. Thus, it is suggested that more effective public policies need to be developed for disease prevention and treatment, allowing for an early diagnosis as soon as the women enter the unit, as well as awareness-raising activities to minimize the risk of contagion. These activities can contribute directly by improving the prison population’s quality of life and, indirectly, by reducing the burden and incidence of syphilis national-wide.
