# Supplementary material for: High prevalence of syphilis in a female prison unit in Northeastern Brazil
Source: Einstein (Sao Paulo). 2020 May 7;18:eAO4978. doi: 10.31744/einstein_journal/2020AO4978 (PMC7206982; doi:10.31744/einstein_journal/2020AO4978)
Supplement: Translation

# Translation: Alta prevalência de sífilis em unidade prisional feminina do Nordeste brasileiro

**Authors:** Mara Ilka Holanda de Medeiros Batista, Marcília Ribeiro Paulino, Kaline Silva Castro, Luiz Alcino Monteiro Gueiros, Jair Carneiro Leão, Alessandra Albuquerque Tavares Carvalho

**Affiliations:** 1 Brasil Universidade Federal de Pernambuco, Recife, PE, Brasil.

**Keywords:** Doenças sexualmente transmissíveis, Infecções, Sífilis/epidemiologia, Saúde da mulher, Prisões, Mulheres, Prisioneiros, Brasil

## Abstract

**Objetivo:**

Determinar a prevalência de sífilis e fatores de risco associados no sistema prisional feminino.

**Métodos:**

Foi realizado estudo transversal com 113 mulheres. A coleta de dados ocorreu em duas etapas: a primeira etapa correspondeu à coleta de sangue para verificar a soropositividade à sífilis e a segunda a um formulário para avaliar situações de risco para infecções sexualmente transmissíveis.

**Resultados:**

A prevalência da doença foi de 22,1% (n=25). Quanto às gestantes, identificou-se prevalência de 28,6%. Foi verificada relação estatisticamente significativa entre a infecção pela sífilis e a história prévia de infecções sexualmente transmissíveis (p=0,04). Entretanto, a maioria das participantes diagnosticadas com a doença desconhecia um histórico de infecção sexualmente transmissível nos últimos 12 meses (n=20/80,0%). O uso de preservativo com parceiros fixos foi considerado fator de proteção (*odds ratio* de 0,76; intervalo de confiança de 95% de 0,68-0,85).

**Conclusão:**

Foi alta a prevalência de sífilis na população carcerária feminina estudada, principalmente entre as gestantes. Medidas preventivas e de tratamento da doença, bem como cuidados pré-natais adequados, podem minimizar o impacto da sífilis nos sistemas prisionais e, consequentemente, melhorar esse indicador de saúde no país.

## INTRODUÇÃO

As doenças sexualmente transmissíveis (DST) têm consequências clínicas e psicológicas importantes aos indivíduos afetados e continuam a apresentar altas taxas globais. Em 2016, foram estimadas 376 milhões de novas infecções, como sífilis, gonorreia, clamídia e tricomoníase, e as taxas variam de acordo com a região.^(1)^ Curiosamente, nos sistemas prisionais, altas taxas de DST são relatadas se comparadas às da população geral. Tais condições podem estar relacionadas a fatores de risco anteriores ao encarceramento, incluindo baixo nível socioeconômico, comportamento sexual de alto risco e uso de drogas injetáveis. Além disso, outros fatores que ocorrem após o encarceramento podem também contribuir para transmissão das DST, como agressão sexual e socialização em grupo.^(2,3)^

A coleta de informações internas sobre DST em unidades prisionais é de grande importância, principalmente se consideramos que os encarceramentos ocorrem por curto período de tempo. O diagnóstico e o tratamento dessas doenças podem cobrir porção considerável da população não tratada no país,^(4)^ além de contribuir para reduzir o risco de contágio. Neste cenário, a sífilis tem sido considerada doença proeminente e é geralmente relatada em sistemas prisionais em todo mundo.^(2,3,5-9)^

A sífilis é uma doença infecciosa causada pela bactéria *Treponema pallidum*, que apresenta implicações clínicas complexas. Sua transmissão ocorre principalmente por meio de atividade sexual desprotegida e transmissão vertical durante a gravidez. Apesar dos principais pontos de inoculação serem os órgãos genitais, áreas extragenitais, como cavidade oral e região anal, também podem ser afetadas.^(10)^

No Brasil, 227.663 casos de sífilis adquirida foram relatadas entre 2010 e 2016, e regiões do Nordeste foram ranqueadas com altas taxas de prevalência (9,3%). Quando avaliada a proporção de doença entre homens e mulheres, verifica-se redução na taxa sexual entre 2010 e 2015, de 1,8 para 1,5 dos casos em homens para cada caso em mulher. Os dados coletados entre os anos de 2005 e 2016 classificaram a Região Nordeste com segunda maior em número de casos relatados em gestantes (21,7%). Além desses dados, entre 2014 e 2015, houve aumento de 32,6% em sífilis adquirida e 20,4% de aumento de sífilis congênita/gestantes.^(11)^

O aumento da incidência de sífilis entre mulheres aponta a necessidade para estratégias diferenciadas em prevenção de infecção.^(12)^ Quando se refere à população feminina, essa categoria é ainda mais vulnerável e requer atenção especial e ações efetivas, com o objetivo de prevenção de doenças e tratamento. Contudo, as características epidemiológicas relacionadas à saúde da mulher encarceradas permanecem largamente desconhecidas.

## OBJETIVO

Determinar a prevalência de sífilis e fatores de risco associados em uma unidade prisional feminina.

## METÓDOS

Estudo descritivo, transversal realizado em 2015 no Centro de Reeducação Feminino na Região Nordeste do Brasil. O total de 343 mulheres correspondia à população prisional, e o estudo universal foi representado por 119 mulheres. Destas, 113 aceitaram participar voluntariamente do estudo, submetendo-se à coleta sanguínea para avaliação de sífilis soropositiva.

Os testes foram realizados por equipe de saúde de unidades prisionais em duas fases. Na primeira fase do estudo, um psicólogo ofereceu aconselhamento para teste rápido, e uma enfermeira ficou responsável pela coleta de sangue para checagem da soropositividade de sífilis. As amostras sanguíneas foram coletadas por meio de punctura digital.

Os testes rápidos utilizados para diagnóstico de sífilis foi o *Rapid Test Dual-Path Plataform* (TR DPP^®^; Bio-Manguinhos, Rio de Janeiro RJ, Brasil). Trata-se de testes de uso único, para detectar anticorpos específicos em amostras de fluido oral, sangue venoso total, punctura digital, e soro humano ou plasma. O teste é baseado em tecnologia imunocromatográfica e utiliza duas rotas. Os resultados reativos são evidência da exposição ao agente infecioso.

A combinação de antígenos *T. pallidum* recombinante ligada à membrana (fase sólida) e à proteína A, conjugada com partículas coloidal de ouro, foi utilizada para detectar anticorpos específicos da doença. A presença de um anticorpo é determinada baseada na aparência de uma linha em dispositivo, que indica reação reativa.

Em uma segunda fase do estudo, os participantes responderam um teste rápido sobre situações de risco para DST proposto pelo Ministério da Saúde (http://formsus.datasus.gov.br/site/formulario.php?id_aplicacao=51700). Com isso, foi possível coletar dados sociodemográficos (idade, cor, estado civil e nível educacional) e os fatores de risco antes e durante o encarceramento, incluindo informações relacionadas ao comportamento sexual (tipo de parceiro, número de parceiros e uso de preservativo) e ao uso de drogas (uso de drogas injetáveis e/ou parceiros de uso de drogas injetáveis). O comportamento de risco foi avaliado baseado na associação de variáveis do estudo abordadas em questionário e do resultado positivo no teste de detecção. As possíveis variáveis relacionadas à soropositividade da doença também foram analisadas.

Os dados foram tabulados utilizando o software (SPSS), versão 21.0. O teste exato de Fisher foi adotado para comparações entre grupos independentes para determinar possíveis associações entre as variáveis analisadas e infecção por sífilis. Valor de p<0,05 foi considerado estatisticamente significante. Para estimar a proporção de indivíduos doentes expostos ou não expostos a fatores de risco, o *odds ratio* foi verificado por meio do cálculo *odds ratio*, com intervalo de confiança de 95%.

Este estudo foi aprovado pelo Comitê de Ética e Pesquisa do Centro Universitário de João Pessoa, protocolo 1.34.953 e CAAE: 48845515.6.00005176. Anterior à confirmação da participação, todos os participantes receberam carta com informações relacionadas ao estudo, sobre os benefícios e os riscos. Aqueles que concordaram em participar, assinaram o Termo de Consentimento.

## RESULTADOS

### Características sociodemográficas

A amostra final foi composta por 113 participantes, com idade entre 18 e 55 anos, com média (± desvio padrão) de idade de 29,8±8,2 anos. Do número total de participantes, 61 (54,0%) autorreferiram-se como mestiças e 72 (63,7%) estavam solteiras. Em relação à educação, 78 (69,1%) eram mulheres e possuíam, no máximo, 7 anos de educação formal (Tabela 1). Sete gestantes participaram no estudo (6,2%).

**Tabela 1:** Características sociodemográficas dos participantes do estudo

Variável	Participantes do estudo
Raça autorreferida	
Mestiça	61 (54,0)
Branca	37 (32,7)
Negra	13 (11,5)
Amarelo	2 (1,8)
Estado civil	
Solteiro	72 (63,7)
Casado/União estável	29 (25,7)
Divorciado	10 (8,8)
Viúvo	2 (1,8)
Escolaridade, anos	
Nenhuma	8 (7,1)
1-3	28 (24,8)
4-7	42 (37,2)
8-11	21 (18,6)
12 ou mais	14 (12,4)

Resultados expressos por n (%).

### Prevalência de sífilis

De 113 participantes, 25 (22,1%) tiveram sorologia positiva para sífilis, o que representou quase um quarto da população de mulheres encarceradas em estudo. Das gestantes, duas (28,6%) foram diagnosticadas com a doença. Todos os resultados que testaram positivo no teste de rápido foram também confirmados pelo teste sanguíneo realizada em hospital de referência da cidade.

### Fatores de risco para sífilis

A prevalência de sífilis e sua associação com fatores de risco estão demonstradas na tabela 2.

**Tabela 2:** Prevalência de sífilis e sua associação com fatores de risco

Fator de risco para sífilis	n	Positivo n (%)	Negativo n (%)	OR	OR IC95%	Valor de p*
Gravidez	113			1,44	0,26-7,93	0,48
Sim		2 (28,6)	5 (71,4)			
Não		23 (21,7)	83 (78,3)			
Parceiro	98			0,83	0,26-2,63	0,48
Homossexual		5 (25,0)	15 (75,0)			
Heterossexual		17 (21,8)	61 (78,2)			
Número de parceiros sexuais nos últimos 12 meses	97			0,85	0,33-2,22	0,47
2 ou mais		12 (24,0)	38 (76,0)			
1		10 (21,3)	37 (78,7)			
Histórico de DST nos últimos 12 meses	113			4,15	1,09-15,72	0,04
Sim		5 (50,0)	5 (50,0)			
Não		20 (19,4)	83 (80,6)			
Uso de preservativos/parceiro fixo	110			0,76	0,68-0,85	0,09
Nunca/ocasionalmente		24 (23,8)	77 (76,2)			
Sempre		0	9 (100,0)			
Uso de preservativos/parceiro casual	90			0,81	0,29-2,23	0,43
Nunca/ocasionalmente		13 (22,0)	46 (78,0)			
Sempre		8 (25,8)	23 (74,2)			
Uso de drogas injetáveis	113			3,62	0,21-60,11	0,39
Sim		1 (50,0)	1 (50,0)			
Não		24 (21,6)	87 (78,4)			
Uso de drogas não injetáveis	113			0,88	0,36-2,14	0,48
Sim		12 (21,1)	45 (78,9)			
Não		13 (23,2)	43 (76,8)			
Histórico de parceiros usuários de droga	113					
Sim		5 (33,3)	10 (66,7)	1,95	0,59-6,35	0,21
Não		20 (20,4)	78 (79,6)			
Consumo frequente de bebidas alcoólicas	113			1,99	0,72-5,49	0,13
Sim		19 (26,0)	54 (74,0)			
Não		6 (15,0)	34 (85,0)			

* Teste estatístico: teste exato de Fisher; estatisticamente significante em p<0,05. IC95%: intervalo de confiança de 95%; OR: *odds ratio*; DST: Doenças Sexualmente Transmissíveis.

Foi observada relação estatisticamente significante entre sífilis e histórico de DST nos últimos 12 meses (p=0,04). Os dados revelaram como fatores de risco: histórico de DST nos últimos 12 meses (OR=4,15; IC95%: 1,09-15,72); uso de drogas injetáveis (OR=3,62; IC95%: 0,21-60,11); relacionamento com parceiros usuários de drogas injetáveis (OR=1,95; IC95%: 0,59-6,35); consumo frequente de bebidas alcoólicas (OR=1,99; IC95%: 0,72-5,49); e gravidez (OR=1,44; IC95%: 0,26-7,93). Uso de preservativo foi considerado fator de proteção para relações com parceiros fixos (OR=0,76; IC95%: 0,68-0,85) e parceiros ocasionais (OR=0,81; IC95%: 0,29-2,23). Relacionamento heterossexual (OR=0,836; IC95%: 0,266-2,63) e não mais do que um parceiro (OR=0,856; IC95%: 0,33-2,22) também foram observados como fatores protetivos. Para todos os fatores mencionados, um intervalo de confiança de 95% foi considerado.

Ao serem descritos os fatores de risco de 113 participantes, 50 (44,2%) deles tiveram relação sexual com dois ou mais parceiros no últimos 12 meses, e 20 (17,6%) mulheres relataram relacionamento homossexual. Para o uso de preservativo, 101 (91,8%) mulheres nunca utilizaram ou usavam ocasionalmente preservativo com seus parceiros fixos, e 54 (65,5%) delas relataram nunca/ocasionalmente utilizar preservativo com parceiros ocasionais. Esses achados demonstram que grande proporção dessas mulheres estava sob risco de ser infectada por DST.

Também é interessante notar que 24 (23,8%) participantes que tiveram relação sexual esporádica ou contínua desprotegida com parceiros fixos foram diagnosticada com sífilis. Entre as mulheres que relataram sempre fazer uso de camisinha, nenhuma desenvolveu a doença.

Sobre o uso de droga, 57 (50,4%) relataram ter utilizado drogas ilícitas não injetáveis. O uso de drogas injetáveis, por outro lado, foi relatado por dois participantes (1,7%). Quando questionadas se seus parceiros eram usuários de drogas injetáveis, 15 (13,3%) disseram que sim. O consumo de bebidas alcoólicas foi prática comum (n=73; 64,6%) entre as participantes.

## DISCUSSÃO

Os resultados observados em nosso estudo por meio de testes rápidos detectaram alta prevalência de sífilis, que foi quase um quarto da população estudada. Tal questão mostra a importância de campanhas de conscientização para população encarcerada na prevenção de doença, detecção e tratamento. É importante ressaltar que falta de conhecimento e infecções não tratadas podem contribuir para aumentar a incidência de sífilis na população geral (não encarcerada).

Marques et al.,^(3)^ também enfatizam que o retorno de indivíduos encarcerados para comunidade possivelmente contribui para aumentar o risco de exposição à doença da população geral.

A sífilis tem sido detectada em diversas unidades prisionais em todo o mundo, com taxas de prevalência variando de 0,7% a 11%.^(2,3,5-9)^ Na prisão estadual de Guanajuato, no México, foi observada prevalência da doença de 0,7%,^(5)^ enquanto em prisões portuguesas encontraram-se altas taxas, como 6,0%.^(3)^ Um estudo no sistema prisional da Venezuela encontrou prevalência sífilis de 6,1%.^(9)^ Em Gana, as taxas de prevalência foram relativamente mais altas (11%).^(5)^ No Brasil, estudo conduzido em Pernambuco identificou prevalência de infecção de 3,92%.^(2)^ Tais taxas geralmente são atribuídas a fatores relacionados à vida sexual e ao uso de drogas injetáveis.

Somente alguns estudos na literatura têm investigado a prevalência de sífilis entre mulheres encarceradas. Esses dados são extremamente importante, considerando que, no Brasil, entre os anos de 2014 e 2015, houve aumento significativo nos casos de sífilis, afetando principalmente mulheres.^(11)^

O estudo de Javanbakht et al.,^(13)^ descreveu a realidade de mulheres de unidade prisional em Los Angeles, nos Estados Unidos, relatando prevalência de sífilis de 1,4%. No Brasil, esses dados são ainda maiores, como apontado em estudo realizado entre mulheres encarceradas em unidade prisional da cidade de São Paulo, com prevalência de 5,7%.^(14)^ Em estudo sobre a realidade de mulheres grávidas encarceradas no Brasil, Domingues et al.,^(15)^ identificaram prevalência de sífilis de 8,7% nesta população. Nosso estudo reuniu mais dados atuais, sugerindo que tal questão teve piora, com taxas de prevalência tão altas quanto 22,1% entre as mulheres e 28,6% entre as gestantes.

Um estudo com gestantes revelou que, entre mulheres que testaram positivo para sífilis, confirmou-se a presença de complicações em mães e fetos, além de sífilis neonatal. Os autores enfatizam que o início da doença durante a gestação merece muita atenção, e seu diagnóstico é essencial para um tratamento efetivo, para prevenir a transmissão vertical.^(16)^ Em estudo retrospectivo, Lafetá et al.,^(17)^ observaram que a sífilis é comumente diagnosticada em estágio tardio entre as gestantes, tanto após o nascimento ou após o processo de curetagem, e a assistência prenatal ou neonatal requer restruturação.

Em nosso estudo, um dos fatores de risco alarmantes foi o uso esporádico de preservativo pela maioria dos participantes. Esse hábito negligente talvez represente o principal fator de contribuição para aumento da incidência da doença no Brasil. Ações educacionais são necessárias para implementação de forma rápida, com o objetivo de reduzir o risco de contágio e incidência de DST.

Estudos transversais têm limitação de não determinarem a relação causa e efeito. Da mesma forma, enfatizamos a necessidade de estudos multicêntricos, para melhorar a representação da realidade do país.

Diversas variáveis podem explicar a potencial alta frequência de sífilis, incluindo o baixo nível socioeconômico da Região Nordeste do Brasil, a escassez de atividades educacionais de prevenção e orientação nas prisões, e o baixo investimento na saúde desta população. De acordo com Domingues et al.,^(15)^ é alta a prevalência de sífilis entre as mulheres encarceradas comparada à da população feminina geral; outros fatores são a baixa qualidade da assistência pré-natal e a alta vulnerabilidade social das mulheres encarceradas.

Outra questão relevante relaciona-se ao tipo de teste utilizado para detectar a doença. Em estudo anterior, Sato et al.,^(18)^ avaliaram teste rápido baseado em técnica imunocromatográfica para detecção de anticorpos contra *T. pallidum*. Os autores observaram valores de sensibilidade e especificidade de 93,7% e 95,2%, respectivamente e sugerem que o teste pode ser aplicado como ferramenta de rastreamento, mas não ser utilizado como critério exclusivo de diagnóstico da infecção. Com isso, a falta de testes complementarem para confirmar os dados pode ser considerada uma limitação, que, contudo, não reduz a natureza dos achados relatados em nosso estudo.

Atualmente, a implementação de iniciativas para prevenir a doença é, em geral, ignorada por órgãos governamentais oficiais devido à falta de informação. Nosso estudo pode contribuir com dados essenciais para planejamento de atividades e reduzir a carga pública relacionada à sífilis em prisões no Nordeste brasileiro e em todo o país.

## CONCLUSÃO

Foi alta a prevalência de sífilis adquirida entre a população feminina encarcerada, especialmente entre gestantes, fato que reforça a necessidade de estabelecimento de medidas urgentes para reduzir a presença da doença e a transmissão em sistemas prisionais. Portanto, sugere-se que políticas públicas mais efetivas sejam desenvolvidas para a prevenção e o tratamento da doença, permitindo um diagnóstico precoce para a mulher assim que admitida na unidade, além de atividades que despertem a conscientização, em termos de melhoria da qualidade de vida da população encarcerada e, indiretamente, pela redução da carga e da incidência nacional da sífilis.
